# Cardiac Sympathetic Nerve Terminals in the Pathogenesis of Takotsubo Syndrome: A Brief Review and Novel Analytical Approach

**DOI:** 10.31083/RCM49787

**Published:** 2026-08-24

**Authors:** Shams Y-Hassan

**Affiliations:** ^1^Department of Cardiology, Karolinska Institutet and Karolinska University Hospital, 141 86 Stockholm, Sweden

**Keywords:** takotsubo syndrome, myocytolysis, myocarditis, neurogenic myocardial stunning, sympathetic denervation

## Abstract

Takotsubo syndrome (TS) is an acute cardiac condition that clinically resembles acute coronary syndrome; however, TS is not caused by acute coronary obstruction. Moreover, TS is characterized by a distinctive regional, often circumferential, and typically reversible left ventricular wall motion abnormality, which can also involve the right ventricle. In addition to the takotsubo sign, the disease has several other characteristic features, including a history of an emotional or physical trigger, repolarization changes on electrocardiography, moderate elevations of biomarkers of myocardial cell necrosis, and histopathological myocardial changes in the form of contraction band necrosis (coagulative myocytolysis) with characteristic time-related evolutionary changes. Several pathophysiologic mechanisms underlying the disease process remain under debate. However, there is evidence of sympathetic nervous system involvement, including local cardiac sympathetic hyperactivation and disruption at cardiac sympathetic nerve terminals, with excessive norepinephrine release and spillover. Hence, the pathogenetic term autonomic neurocardiogenic syndrome (ANCA), with or without a takotsubo phenotype, has been introduced. For decades, the histopathological cardiac lesions of focal or multifocal coagulative myocytolysis and focal or multifocal “myocarditis” in TS/ANCA syndrome have been described as diagnostic terms in the medical literature. However, the circumferential and multifocal nature of this disease, along with the characteristic time-related histopathological changes and its presence within normal myocardium, provide further evidence that TS/ANCA syndrome is a disorder of the cardiac sympathetic nerve terminals. Therefore, this review presents evidence supporting the previously unquestioned pathogenetic mechanism, with a focus on the specific role of cardiac sympathetic nerve terminals in this syndrome.

## 1. Introduction

Takotsubo syndrome (TS) is an acute cardiac condition that clinically mimics acute coronary syndrome; however, TS typically presents without obstructive coronary artery disease in most cases. TS is characterized by a unique regional, often circumferential, and typically reversible left ventricular wall motion abnormality (LVWMA), which can also sometimes occur in the right ventricular wall [1,2,3]. In addition to the classical takotsubo-shaped left ventricle during systole, TS has several other characteristic features, which include a history of an emotional or physical trigger; repolarization changes on electrocardiography (ECG); modest elevations in biomarkers of myocardial cell necrosis; characteristic histopathological changes in the form of contraction band necrosis (also known as coagulative myocytolysis or myofibrillar degeneration) [1,2,3,4,5]. Moreover, evidence for the involvement of the sympathetic nervous system in the pathogenesis of TS is abundant, including hyperactivation of the cardiac sympathetic nervous system, with excessive norepinephrine release and spillover at cardiac sympathetic nerve terminals [6,7]. Thus, the pathogenetic term autonomic neurocardiogenic syndrome (ANCA), with or without a takotsubo phenotype, was introduced [7]. Therefore, this review applies the diagnostic term of TS, and the pathogeneticterm ANCA syndrome (with or without takotsubo phenotype) interchangeably. Cardiac coagulative myocytolysis is a histopathological term used to describe a pathological process in which cardiac myocytes undergo irreversible hypercontraction with breakdown of the contractile apparatus, ranging from coagulated, hypercontracted sarcomeres to complete disruption of the myofibrils with granular degeneration [8,9]. For decades, the cardiac histopathological feature of focal or multifocal coagulative myocytolysis and sometimes, incorrectly, focal or multifocal “myocarditis”, has been described in the medical literature, both in diagnostic terminology and in publication titles, well before and even during the early takotsubo era [10,11,12,13]. Thus, to our knowledge, this manuscript provides evidence for the first time that the multifocal nature of this disease, with its circumferential distribution interspersed with normal viable myocardium, further supports the involvement of cardiac sympathetic nerve terminals in the pathogenesis of TS/ANCA syndrome.

## 2. Pathophysiology of TS/ANCA Syndrome

The following are among the most debated pathogenic mechanisms of TS [6,14,15,16,17]: (A) Hyperactivation of the sympathetic nervous system, including local cardiac sympathetic hyperactivation and disruption, with excessive norepinephrine release/spillover at cardiac sympathetic nerve terminals [6,7]. (B) Epinephrine-induced switching in stimulus trafficking of β_2_-adrenoreceptors [18]. (C**)** Blood-borne catecholamine-induced myocardial toxicity [15,17]. (D) Coronary ischemia caused by aborted myocardial infarction (MI) in patients with a long wrap-around left anterior descending artery [19], multivessel coronary artery spasm [20], or coronary microvascular dysfunction [21]. (E) Left ventricular outflow tract obstruction [15,17]. (F) Transiently increased ventricular afterload induced by a catecholamine surge [22]. (G) Estrogen deficiency, nitrosative stress, inflammation, and cardiac macrophages [17]. Evidence challenging the last six hypotheses listed above is substantial and has been discussed previously [6,7,13,23].

In contrast, robust evidence supports the hypothesis that the local cardiac sympathetic nervous system is involved in the pathogenesis of TS [6,7]. Among the most important supporting observations is that various brain diseases and injuries, including intracranial hemorrhage, subarachnoid hemorrhage (SAH), stroke, and epilepsy, may trigger TS [24,25]. Structural and functional brain changes have been reported in patients with TS [26,27,28,29]. The organized and systematic LVWMA in TS appears to follow the anatomical pattern of sympathetic innervation from the left and right stellate and caudal ganglia [30,31]. Studies using ^123^I-metaiodobenzylguanidine (^123^I-MIBG) to image cardiac sympathetic nerve function have demonstrated that patients with TS exhibit cardiac sympathetic denervation in regions with LVWMA, characterized by decreased regional uptake and increased ^123^I-MIBG washout [32,33,34,35]. Additional evidence for cardiac sympathetic dysfunction in TS is provided elsewhere [5,6,7].

A previously undebated observation regarding the involvement of cardiac sympathetic nerve terminals in the pathogenesis of TS/ANCA syndrome is the multifocal nature of the histopathological features associated with the disease, described as “focal/multifocal coagulative myocytolysis” or myocarditis. This perspective provides a novel interpretation of the pathophysiology of TS/ANCA syndrome. The arguments are presented in detail, alongside supporting illustrative figures.

## 3. Multifocal Nature of Cardiac Coagulative Myocytolysis or “Myocarditis” in the Pathogenesis of TS/ANCA Syndrome

### 3.1 Historic Background

Coagulative myocytolysis, also known as contraction band necrosis (a form of myocardial necrosis, also termed catecholamine necrosis), is a histopathological cardiac finding characterized by irreversible hypercontraction of the cardiac myocyte, breakdown of the contractile apparatus, markedly thickened Z-lines, and extremely short sarcomeres [8,9]. The terms “focal/multifocal myocytolysis” and “focal/multifocal myocarditis” have appeared in the literature as diagnostic labels and in publication titles for decades, well before the era of TS [10,11,12]. In 1955, Schlesinger and Reiner [10] described focal myocytolysis of the heart as miliary cardiac lesions characterized by loss of the muscular syncytium, preservation of the stroma, absence of an inflammatory reaction, and eventual fibrosis. The authors noted that focal myocytolysis, as it appears at the borders of cardiac infarcts, had been recognized as early as 1904 [10]. According to these investigators, other authors described focal myocytolysis under various designations, including acute miliary infarctions, focal necrotizing “myocarditis” without interstitial infiltration, and sarcolytic myocardiosis [10]. “Zenker's necrosis” has also been used as an alternative term [36]*.* In 1966, Van Vliet et al. [11] reported “focal myocarditis” associated with pheochromocytoma. The investigators found histological signs of “active myocarditis” in 15 (58%) of the 26 patients who died of pheochromocytoma. The active “myocarditis” observed in pheochromocytoma was believed to be catecholamine-induced because the “myocarditis” shared the same histopathological features as the “active myocarditis” induced in rats after one or two subcutaneous L-norepinephrine injections. Subsequently, Jepson et al. [12] reported two cases, in which pheochromocytoma was discovered postmortem following unexpected postoperative death. Histological examination also showed focal myocardial degeneration with necrosis and inflammatory cell infiltration, suggesting “focal myocarditis”. Baratella et al. [37] described a case of focal myocardial necrosis with lymphocytic infiltration on endomyocardial biopsy, diagnosed as “acute lymphocytic myocarditis” in a patient with pheochromocytoma. These reports support the findings described by Van Vliet et al. [11]. High catecholamine doses can cause myocardial necrotic lesions accompanied by “inflammation” and subsequent fibrous scarring, a phenomenon also observed in animal models [38]. The clinical counterpart is observed in patients dying after prolonged norepinephrine treatment and in those dying with pheochromocytoma, as discussed above [38]. Over the last two decades, it has been well-documented that catecholamine administration, whether therapeutic, accidental, or occurring in conditions associated with markedly elevated catecholamine levels, such as pheochromocytoma and paraganglioma, may trigger TS—a condition frequently associated with cardiac histopathological findings of contraction band necrosis (coagulative myocytolysis) [39,40,41,42].

### 3.2 Evolutionary Histopathologic Mechanism of Multifocal Coagulative Myocytolysis or “Myocarditis” in TS/ANCA Syndrome

**Fig. 1.**
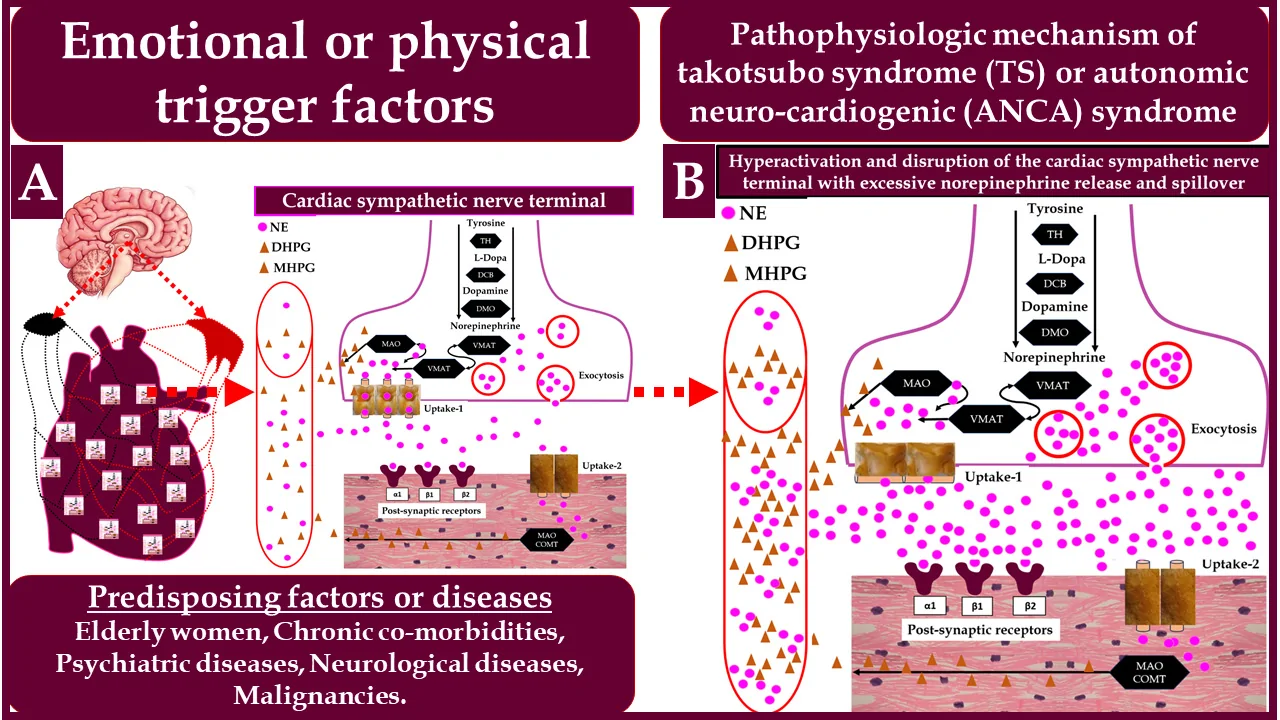
**Pathophysiologic mechanism of takotsubo syndrome (TS) or autonomic neurocardiogenic syndrome (ANCA) at the cardiac sympathetic nerve terminals**. (A) The brain and the sympathetic nerve supply of the heart, including the cardiac sympathetic nerves, end at the cardiac sympathetic nerve terminals. (B) In a predisposed patient, an emotional or physical trigger may lead to hyperactivation of the cardiac sympathetic nerves and disruption at the cardiac sympathetic nerve terminals, which could result in excessive norepinephrine (NE) release/spillover. The consequences of NE spillover are described in the main manuscript text. DHPG, dihydroxyphenylglycol; MHPG, 3-methyl-4-hydroxyphenylglycol.

**Fig. 2.**
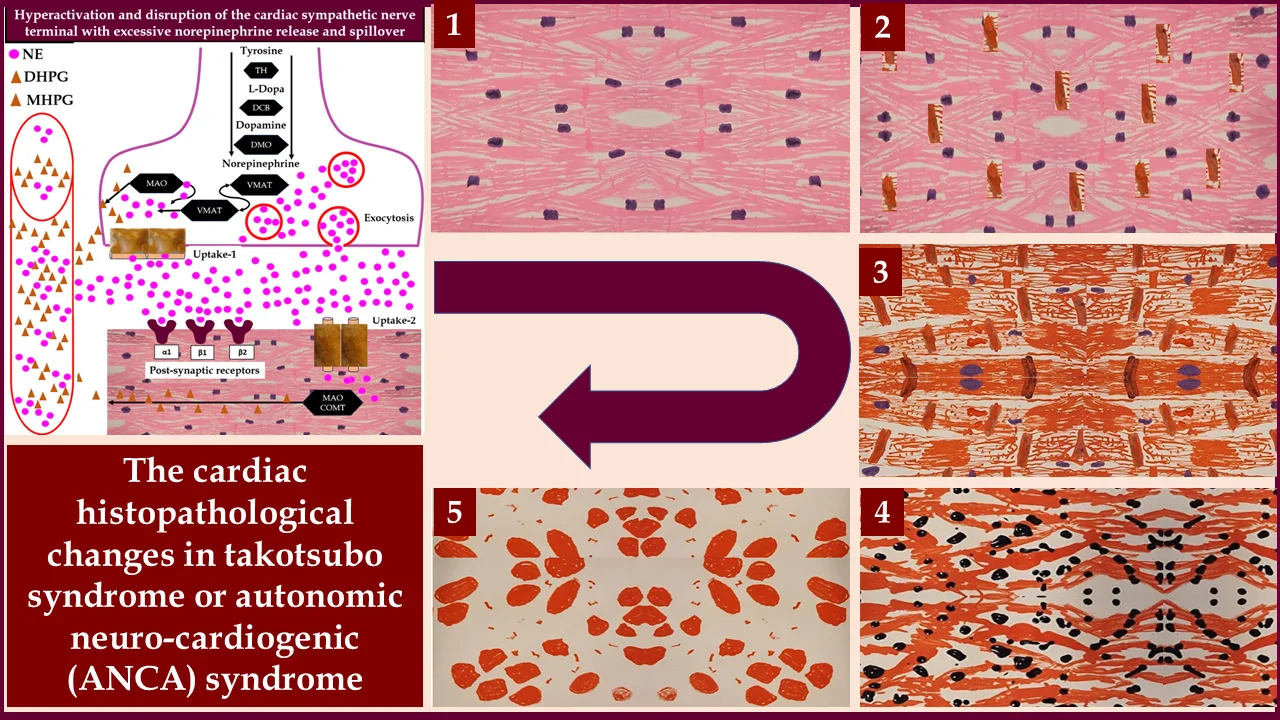
**Evolutionary stages of the cardiac histopathological changes observed in severe cases of TS/ANCA syndrome**. (1) Normal myocardium; (2) myocardium with contraction bands; (3) myocardium with contraction band necrosis (CBN; coagulative myocytolysis); (4) myocardium with CBN and mononuclear cell infiltration (myocarditis-like changes); (5) resolution of the inflammatory response with healing and scar formation. Notably, the myocarditis-like changes shown in (4) are proposed to occur after myocardial necrosis has occurred, as is seen in (3). For further details on the histopathological features, please see Figs. 3,4.

The histopathological features of the cardiac lesions in TS/ANCA syndrome are the main reason these cases have been reported in the literature under the headings of focal/multifocal myocytolysis or “myocarditis” [10,11,12,13]. Notably, both focal myocytolysis and focal “myocarditis” in TS/ANCA syndrome share the same underlying pathophysiology but represent different temporal stages of the same disease process (Figs. 1,2). Baroldi et al. [8,43] described these stages in patients with MI, in whom different stages of contraction band necrosis were observed beyond the infarcted regions. Similar time-related evolutionary cardiac histopathological changes have been reported in patients with SAH and in animal studies [44]. The local pathological process begins at the cardiac sympathetic nerve terminal, where large amounts of norepinephrine are released into the myocardium due to cardiac sympathetic hyperactivation and disruption (Fig. 1) [45,46,47]. This results in the opening of calcium channels, with subsequent cellular influx of Ca^2+^ and efflux of K^+^ [48,49]. This process causes actin and myosin filaments to interact; relaxation does not occur unless the calcium channels close. If high norepinephrine levels persist, the calcium channels remain open, leading to cell death while the myocyte is in an excessively contracted state [48,49]. The histopathological change at this stage (Fig. 2) is called contraction band necrosis (coagulative myocytolysis). This change is focal because the process follows the distribution of the cardiac sympathetic nerve terminals, hence, the diagnostic term of “focal myocytolysis”. The same pathological process may occur at many cardiac sympathetic nerve terminals along the course of the cardiac sympathetic nerves, leading to the term multifocal myocytolysis. The next phase in the histopathological evolution of the disease process is characterized by myocardial cell necrosis, which elicits a mononuclear cell response [8,43,44]. The cardiac lesion at this stage is interpreted as “myocarditis”, hence the designation “focal/multifocal myocarditis” (Fig. 2). Notably, this is not primary myocarditis but a secondary process, more accurately described as having “secondary myocarditis-like” histopathological features [13]. The phase of mononuclear cell infiltration, which is interpreted as “myocarditis”, always follows the occurrence of myocardial cell necrosis. Consequently, focal myocytolysis and focal “myocarditis” represent distinct evolutionary stages of histopathological change within the same disease process (Fig. 2). These are sometimes described as multifocal because cardiac lesions can appear at numerous sympathetic nerve terminals along the cardiac sympathetic nerves. Interestingly, the multifocal nature of the disease is also evident on cardiac magnetic resonance imaging, manifesting as multifocal late gadolinium enhancement corresponding to regions of left ventricular ballooning, typically observed in more severe forms of TS/ANCA syndrome [13].

### 3.3 Multifocal Coagulative Myocytolysis is a Non-Ischemic Lesion Typically Observed Adjacent to Intramyocardial Nerves

One could argue that the focal or multifocal nature of this disease may be attributable to MI; however, MIs are usually monofocal. Furthermore, the histopathological process leading to coagulative myocytolysis is distinct from that in ischemic infarct necrosis, in which myocardial cells die during relaxation. The myocardial cells in coagulative myocytolysis die due to prolonged sarcomere hypercontraction [8,43,44]. The distribution of these cardiac lesions appears unrelated to blood supply, as these lesions are found adjacent to normal capillaries. Greenhoot and Reichenbach [44] also demonstrated the multifocal nature of these cardiac lesions in both patients who died from SAH and an animal study where the midbrain reticular formation was stimulated. The authors thoughtfully documented the histologic evolution of the cardiac lesions and correlated these findings with patient survival time. The authors further observed that the most severe lesions were located adjacent to intramyocardial nerves. Thus, the multifocal nature of these cardiac lesions further supports the involvement of cardiac sympathetic nerve terminals in the pathogenesis of TS or ANCA syndrome.

### 3.4 Circumferential Pattern of Multifocal Coagulative Myocytolysis or “Myocarditis” Amidst Normal Myocardium

**Fig. 3.**
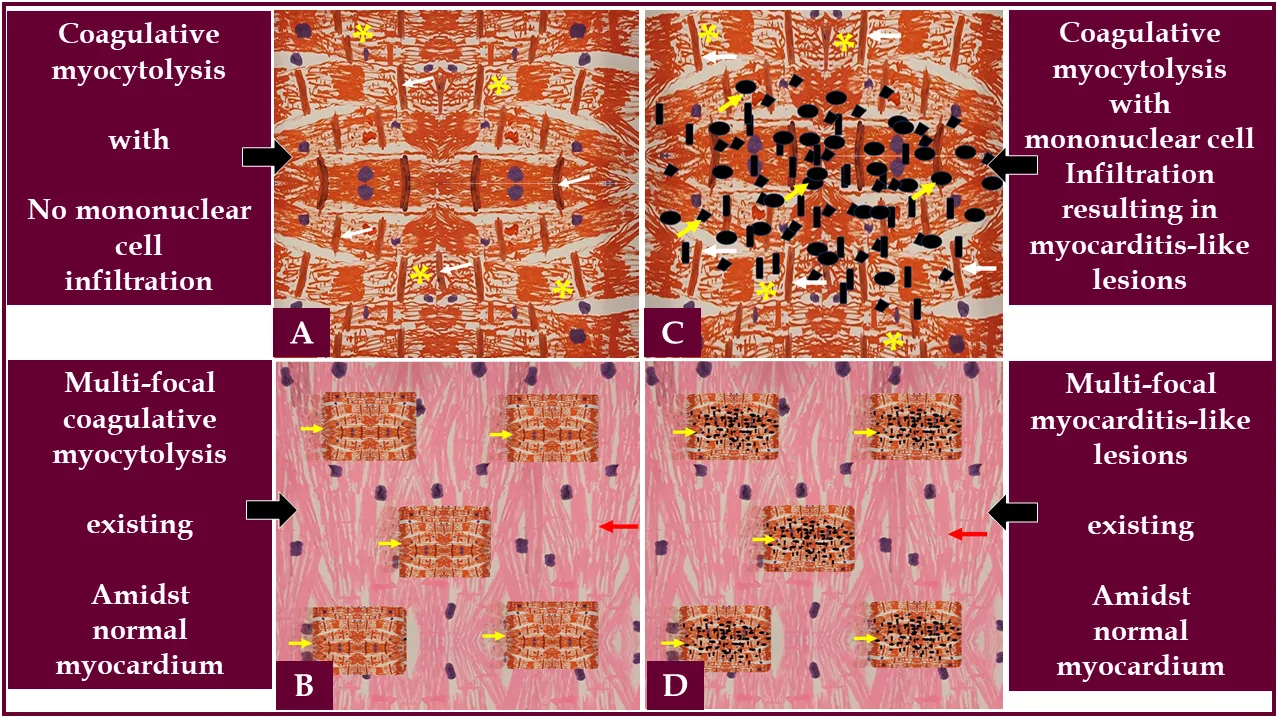
**Time-related multifocal coagulative myocytolysis and “myocarditis-like” lesions amidst normal myocardium**. (A) Cardiac coagulative myocytolysis lesion with contraction band necrosis (white arrows) and intervening granularity (yellow asterisks). (B) Multifocal cardiac coagulative myocytolysis lesions (yellow arrows) amidst normal myocardium (red arrow). (C) Coagulative myocytolysis lesion infiltrated by mononuclear cells (yellow arrows), resulting in a “myocarditis-like” lesion. (D) Multifocal “myocarditis-like” lesions (yellow arrows) amidst normal myocardium (red arrow).

**Fig. 4.**
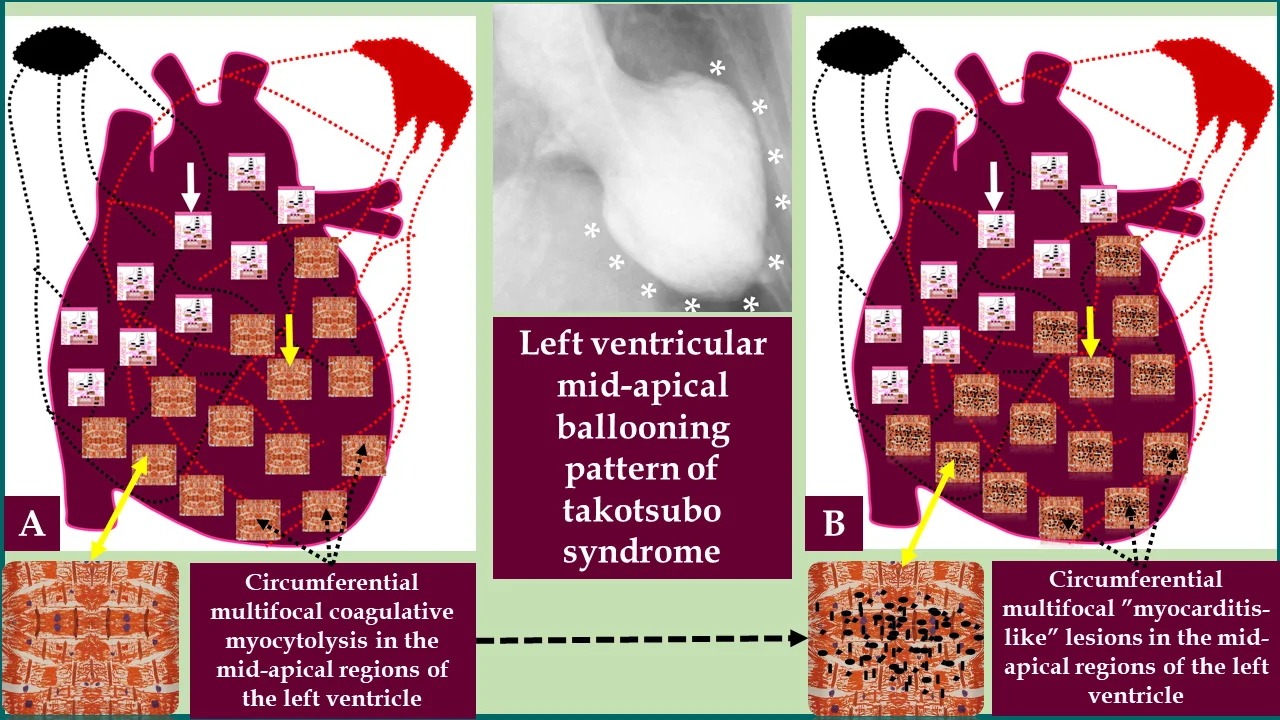
**Multifocal nature and circumferential distribution of coagulative myocytolysis and “myocarditis-like” lesions, in a pattern identical to the regional circumferential ballooning pattern of TS**. (A) Multifocal and circumferential distribution of coagulative myocytolysis (yellow arrows) in the mid-apical segments of the left ventricle, in a pattern identical to the mid-apical ballooning pattern seen in the TS case shown in the center of the figure. The cardiac sympathetic nerve terminals in the basal segments are normal (white arrow). (B) Multifocal and circumferential distribution of the “myocarditis-like” lesions (yellow arrows), which follow the same pattern as in panel (A). The only difference between the lesions in (A and B) is the infiltration of the coagulative myocytolysis lesions by mononuclear cells, resulting in the “myocarditis-like” lesions shown in (B).

Another characteristic feature of these multifocal cardiac lesions is their circumferential pattern, interspersed within an otherwise normal, viable myocardium. This has been described in a publication by Düren and Becker [50]. The authors reported two cases under the title “focal myocytolysis mimicking the ECG pattern of transmural antero-septal MI”. One patient had a cerebral infarction, and the other had a cerebral tumor. In both cases, the autopsy showed no signs of MI; however, the autopsy confirmed that the ECG changes were related to focal myocytolysis of the myocardium, where signs of both contraction band necrosis and mononuclear cell infiltration were observed. Moreover, multiple foci of such lesions were identified at cardiac autopsy. Among viable myocardial cells, a small cluster of degenerating cells was observed and was engulfed by mononuclear cells. The greatest density of lesions was noted in the apical segments of the left ventricle, where the lesions formed a circumferential pattern reminiscent of left ventricular apical ballooning seen in patients with TS. The findings in these two cases further precisely illustrate the multifocal nature of the disease, which produces histopathological changes of multifocal myocytolysis with “secondary myocarditis-like” changes and follows the course of the cardiac sympathetic nerves. The presence of cardiac myocytolytic foci within normal myocardium has also been clearly described in another case reported by Sakamoto et al. [51]. The authors described a 74-year-old woman with SAH and ECG repolarization changes, including abnormal Q waves, ST-segment elevations, and T-wave inversions. Postmortem examination revealed widespread focal myocytolytic cardiac lesions; however, no macroscopic evidence of coronary artery disease or MI was observed. The cardiac lesions were described as multifocal myocytolysis with a scant mononuclear cellular infiltrate and were present throughout the circumference of the left ventricle. The foci of myocytolysis were surrounded by normal myocardial cells and were not related to the vascular distribution. The histopathological morphology of coagulative myocytolysis, with or without myocarditis-like features, the associated multifocal nature, and the presence among normal myocardium are illustrated in Figs. 3,4. The distribution of coagulative myocytolysis in a circumferential pattern in the mid-apical region is identical to the circumferential pattern of the LVWMA observed in TS and is clearly demonstrated in Fig. 4. These two figures illustrate the observations of Düren and Becker [50] and Sakamoto et al. [51].

Consequently, the multifocal nature of cardiac coagulative myocytolysis, or “secondary myocarditis-like” lesions, provides additional evidence that TS/ANCA syndrome is a disease of the cardiac sympathetic nerve terminals, which follow the distribution of the cardiac sympathetic nerves.

## 4. Conclusion

Compelling evidence indicates that cardiac sympathetic hyperactivation and disruption at cardiac sympathetic nerve terminals, with excessive norepinephrine release and spillover, contribute to the pathogenesis of TS. Therefore, the pathogenetic term of ANCA syndrome is also used. The histopathological cardiac lesions of this disease have long been described under the diagnostic terms focal/multifocal coagulative myocytolysis and focal/multifocal “myocarditis”. The multifocal nature, the characteristic time-dependent histopathological evolution (with myocarditis-like changes consistently following coagulative myocytolysis), the circumferential distribution pattern, and the presence of focal lesions within otherwise normal, viable myocardium and in proximity to nerves further support the hypothesis that TS/ANCA syndrome is a disease of the cardiac sympathetic nerve terminals that follows the path of cardiac sympathetic nerves. This novel analytical framework for the pathophysiology of TS/ANCA syndrome is presented, discussed, and illustrated with informative figures.

## Abbreviations

ANCA, autonomic neurocardiogenic syndrome; ECG; electrocardiography; LVWMA, left ventricular wall motion abnormality; MI, myocardial infarction; SAH, subarachnoid hemorrhage; TS, takotsubo syndrome; ^123^I-MIBG, ^123^I-metaiodobenzylguanidine.
